# Molecular analysis of Salmonella paratyphi A from an outbreak in New Delhi, India.

**DOI:** 10.3201/eid0403.980348

**Published:** 1998

**Authors:** K L Thong, S Nair, R Chaudhry, P Seth, A Kapil, D Kumar, H Kapoor, S Puthucheary, T Pang


clinical presentation of the patients, response to trivalent botulinum antitoxin, and isolation of toxigenic *C. butyricum* from one of the consumed food articles strongly suggest that the outbreak was caused by food contaminated with toxigenic *C. butyricum*.

Neurotoxigenic *C. butyricum* was first reported in 1986 in two cases of infant botulism in Rome (6). Recently, neurotoxigenic *C. butyricum* was isolated from the food implicated in an outbreak of clinically diagnosed type E botulism in China (7). In this outbreak, it appears that sevu, because of improper storage, was contaminated with the spores of *C. butyricum*, which subsequently germinated and produced toxin. To the best of our knowledge, this is the first report of neurotoxigenic *C. butyricum* causing foodborne botulism in India.

The changing epidemiology of foodborne disease as highlighted in this report calls for improved surveillance, including the development of new technology for identifying outbreaks.

We thank Alison East, Institute of Food Research, Reading Laboratory, United Kingdom, for supplying *E. coli* clones with BONT gene for PCR; Pradeep Seth, professor and head, Department of Microbiology, All India Institute of Medical Sciences for facilities provided; Biomed Warsaw, Poland, for polyvalent botulinum antitoxin; and the medical and paramedical staff of Civil Hospital, Ahemdabad.

**Rama Chaudhry,* Benu Dhawan,* Dinesh Kumar,* Rajesh Bhatia,† J.C Gandhi,‡ R.K. Patel,§ and B.C. Purohit§**

All India Institute of Medical Sciences, New Delhi, India; †National Institute of Communicable Diseases, Delhi, India; ‡Health Medical Services and Medical Education (H.S.), Gandhi Nagar, India; and §Civil Hospital, Ahemdabad, Gujrat, India

### References

1. Hatheway CL. Botulism. In: Balows A, Hausler WJ, Lennette EH, editors. Laboratory diagnosis of infectious diseases: principles and practice. New York: Springer-Verlag: 1988. p. 111-33.
2. McCroskey LM, Hatheway CL, Fenicia L, Pasolini B, Aureli P. Characterization of an organism that produces type E botulinal toxin but which resembles *Clostridium butyricum* from the feces of an infant with type E botulism. J Clin Microbiol 1986;23:201-2.
3. Campbell KD, Collins MD, East AK. Gene probes for identification of the Botulinal Neurotoxin gene and specific identification of neurotoxin types B.E. and F.J. Clin Microbiol 1993;31:2255-62.
4. Hatheway CL. *Clostridium botulinum* and other clostridia that produce botulinum neurotoxin. In: Hauschild AHW, Dodds KL, editors. *Clostridium botulinum*—ecology and control in foods. New York: Marcel Dekker, Inc.; 1992. p. 3-20.
5. Woodruff BA, Griffin PM, McCroskey LM, Smart JF, Wainwright RB, Bryant RG, et al. Clinical and laboratory comparison of botulism from toxin types A, B, and E in the United States, 1975-1988. J Infect Dis 1992;166:1281-6.
6. Aureli PK, Fenicia L, Pasolini B, Gianfranceschi M, McCroskey LM, Hatheway CL**.** VII. Two cases of type E infant botulism caused by neurotoxigenic *Clostridium butyricum* in Italy. J Infect Dis 1986;154:207-11.
7. Meng X, Karasawa T, Zou K, Kuang X, Wang X, Lu C. et al. Characterization of a neurotoxigenic *Clostridium butyricum* strain isolated from the food implicated in an outbreak of food-borne type E botulism. J Clin Microbiol 1997;35:2160-2.

### Molecular Analysis of *Salmonella paratyphi* A From an Outbreak in New Delhi, India

**To the Editor:** In the context of emerging infectious diseases, enteric fever caused by *Salmonella paratyphi* A deserves increased attention and vigilance, although its severity is often milder than that of *S. typhi* disease. Outbreaks associated with this organism are exceedingly rare but have recently been reported in India (1) and Thailand. In India, the first reported outbreak of disease associated with *S. paratyphi* A (1) provided an opportunity to study the molecular epidemiology of infection caused by this organism.

A total of 18 human blood isolates of *S. paratyphi* A, 13 from the outbreak in New Delhi, India (from September to October 1996) (1) and 5 sporadic isolates from cases unrelated to the outbreak, were used in this study. A total of 36 culture-positive cases were detected during the 6-week outbreak. All strains were phage type 1 and were sensitive to all antibiotics tested. Isolates were analyzed by ribotyping and pulsed-field gel electrophoresis (PFGE) (2,3). PFGE/ribotype profiles were assigned arbitrary designations and analyzed by defining a similarity (Dice) coefficient, F (3), where F = 1.0 indicates complete pattern identity and F = 0, complete dissimilarity.

The five sporadic isolates of *S. paratyphi* A gave PFGE patterns following *Xba*I (5'-TCTAGA-3') digestion that were unique and distinctly different, with differences of 8 to 12 bands (F = 0.63-0.70). In contrast, the 13 outbreak isolates shared only four closely related PFGE patterns differing only in 1 to 6 bands (F = 0.8-1.0). Among the outbreak strains, two distinct clones were observed, X1 and X2, which differed by 5 to 6 bands. Furthermore, outbreak isolates X3 and X4 were closely related to X1, differing by four and three DNA fragments, respectively. Similar results were obtained after digestion with a second restriction endonuclease, *Spe*I (5'-ACTAGT-3'; pattern designation S). Although fewer bands were seen compared to PFGE, ribotyping of these isolates using *Spe*I-digested genomic DNA largely confirmed the PFGE results in that the sporadic isolates gave unique profiles and only three closely related ribotype profiles were detected among the outbreak isolates. Two Malaysian isolates of *S. paratyphi* A included for comparison gave patterns very different from the Indian isolates by both PFGE (F = 0.44-0.65) and ribotyping. Also, it was determined that isolates A-117 (X1/S1) and A-123 (X2/S2) belonged to the index cases and that, as the outbreak progressed, other patterns (X3/S3 and X4/S4), which differed from the original patterns by one to four bands, appeared during weeks 2 to 3 of the outbreak. Notably, patterns X1 and X2 reappeared at the end of the outbreak.

Although molecular analysis of *S. typhi* and *S. paratyphi* B by ribotyping (2,4) and PFGE (3) has been reported, to the best of our knowledge the present study is the first performed with *S. paratyphi* A. The data obtained agree with those observed for *S. typhi* (3) in that outbreak isolates are more clonal and limited in diversity, whereas sporadic isolates are more diverse genetically and belong to unrelated clones. According to the criteria of Tenover et al. (5), it seems likely that the present outbreak was associated with two distinct clones/strains of *S. paratyphi* A (X1/S1 and X2/S2) that are related (5) but have distinct PFGE profiles. This observation is perhaps not surprising given the fact that both clones are phage type 1 and that contaminated potable water was incriminated in the outbreak (1). The PFGE results were largely confirmed by ribotyping, although this technique appears to be slightly less sensitive and discriminating in that fewer bands were seen and the differences between outbreak isolates were much less obvious.

We thus conclude that the outbreak in New Delhi, India, was caused by two related but distinct clones of *S. paratyphi* A. There also appears to be substantial genetic diversity among *S. paratyphi* A strains as the Malaysian isolates were very different from those from India. The data also suggested minor genetic changes among the *S. paratyphi* A isolates during the 2-month outbreak. This observation agrees with the high mutation rates noted among pathogenic *Salmonella* spp. (6) and the plasticity of the genome of salmonellae associated with enteric fever (7). How these changes affected the biologic behavior of these isolates will be the subject of further study. Our study reaffirms the usefulness of PFGE and ribotyping in the molecular typing and discrimination of individual *Salmonella* isolates for epidemiologic investigations.

**Kwai-Lin Thong,* Satheesh Nair,* Rama Chaudhry,† Pradeep Seth,† Arti Kapil,† Dinesh Kumar,† Hema Kapoor,‡ Savithri Puthucheary,* and Tikki Pang***

*University of Malaya, Kuala Lumpur, Malaysia; †All India Institute of Medical Sciences, New Delhi, India; and ‡Safdarjang Hospital, New Delhi, India

### References

1. Kapil A, Sood S, Reddaiah VP, Das B, Seth P. Paratyphoid fever due to *Salmonella enterica* serotype paratyphi A. Emerg Infect Dis 1997;3:407.
2. Pang T, Altwegg M, Martinetti G, Koh CL, Puthucheary SD. Genetic variation among Malaysian isolates of *Salmonella typhi* as detected by ribosomal RNA gene restriction patterns. Microbiol Immunol 1992;36:539-43.
3. Thong KL, Cheong YM, Puthucheary S, Koh CL, Pang T. Epidemiologic analysis of sporadic and outbreak *Salmonella typhi* isolates by pulsed field gel electrophoresis. J Clin Microbiol 1994;32:1135-41.
4. Ezquerra E, Burnens A, Jones C, Stanley J. Genotypic typing and phylogenetic analysis of *Salmonella paratyphi* B and *S. java* with IS200. J Gen Microbiol 1993;139:2409-14.
5. Tenover FC, Arbeit RD, Goering RV, Mickelson PA, Murray BE, Persing DH, et al. Interpreting chromosomal DNA restriction patterns produced by pulsed field gel electrophoresis: criteria for bacterial strain typing. J Clin Microbiol 1995;33:2233-9.
6. Leclerc JE, Li B, Payne WL, Cebula TA. High mutation frequencies among *Escherichia coli* and *Salmonella* pathogens. Science 1996;274:1208-11.
7. Liu SL, Sanderson KE. Highly plastic chromosomal organization in *Salmonella typhi*. Proc Natl Acad Sci U S A 1996;93;10303-8.

### Unrecognized Ebola Hemorrhagic Fever at Mosango Hospital during the 1995 Epidemic in Kikwit, Democratic Republic of the Congo

**To the Editor:** We report here the clinical description of a hemorrhagic syndrome observed

